# Therapeutic S100A8/A9 blockade inhibits myocardial and systemic inflammation and mitigates sepsis-induced myocardial dysfunction

**DOI:** 10.1186/s13054-023-04652-x

**Published:** 2023-09-29

**Authors:** Gabriel Jakobsson, Praveen Papareddy, Henrik Andersson, Megan Mulholland, Ravi Bhongir, Irena Ljungcrantz, Daniel Engelbertsen, Harry Björkbacka, Jan Nilsson, Adrian Manea, Heiko Herwald, Marisol Ruiz-Meana, Antonio Rodríguez-Sinovas, Michelle Chew, Alexandru Schiopu

**Affiliations:** 1 Department of Translational Medicine, Lund University, Lund, Sweden; 2 Department of Clinical Sciences Lund, Lund University, Lund, Sweden; 3 Department of Anaesthesia and Intensive Care, Biomedical and Clinical Sciences, Linköping University, Linköping, Sweden; 4 Department of Clinical Sciences Malmö, Lund University, Lund, Sweden; 5 Nicolae Simionescu Institute of Cellular Biology and Pathology, Bucharest, Romania; 6 Cardiovascular Diseases Research Group, Vall d’Hebron Institut de Recerca, Vall d’Hebron Hospital Universitari, Barcelona, Spain; 7 Centro de Investigación Biomédica en Red de Enfermedades Cardiovasculares, Instituto de Salud Carlos III, Madrid, Spain; 8 Department of Internal Medicine, Skane University Hospital, Lund, Sweden; 9 Cardiac Inflammation Research Group, Clinical Research Center, 91:12, Jan Waldenströms Gata 35, 21 428 Malmö, Sweden

**Keywords:** S100A8/A9, Sepsis-induced myocardial dysfunction, Endotoxemia, Mitochondrial function, Inflammation, Neutrophils

## Abstract

**Background and Aims:**

The triggering factors of sepsis-induced myocardial dysfunction (SIMD) are poorly understood and are not addressed by current treatments. S100A8/A9 is a pro-inflammatory alarmin abundantly secreted by activated neutrophils during infection and inflammation. We investigated the efficacy of S100A8/A9 blockade as a potential new treatment in SIMD.

**Methods:**

The relationship between plasma S100A8/A9 and cardiac dysfunction was assessed in a cohort of 62 patients with severe sepsis admitted to the intensive care unit of Linköping University Hospital, Sweden. We used S100A8/A9 blockade with the small-molecule inhibitor ABR-238901 and S100A9^−/−^ mice for therapeutic and mechanistic studies on endotoxemia-induced cardiac dysfunction in mice.

**Results:**

In sepsis patients, elevated plasma S100A8/A9 was associated with left-ventricular (LV) systolic dysfunction and increased SOFA score. In wild-type mice, 5 mg/kg of bacterial lipopolysaccharide (LPS) induced rapid plasma S100A8/A9 increase and acute LV dysfunction. Two ABR-238901 doses (30 mg/kg) administered intraperitoneally with a 6 h interval, starting directly after LPS or at a later time-point when LV dysfunction is fully established, efficiently prevented and reversed the phenotype, respectively. In contrast, dexamethasone did not improve cardiac function compared to PBS-treated endotoxemic controls. S100A8/A9 inhibition potently reduced systemic levels of inflammatory mediators, prevented upregulation of inflammatory genes and restored mitochondrial function in the myocardium. The S100A9^−/−^ mice were protected against LPS-induced LV dysfunction to an extent comparable with pharmacologic S100A8/A9 blockade. The ABR-238901 treatment did not induce an additional improvement of LV function in the S100A9^−/−^ mice, confirming target specificity.

**Conclusion:**

Elevated S100A8/A9 is associated with the development of LV dysfunction in severe sepsis patients and in a mouse model of endotoxemia. Pharmacological blockade of S100A8/A9 with ABR-238901 has potent anti-inflammatory effects, mitigates myocardial dysfunction and might represent a novel therapeutic strategy for patients with severe sepsis.

**Graphical Abstract:**

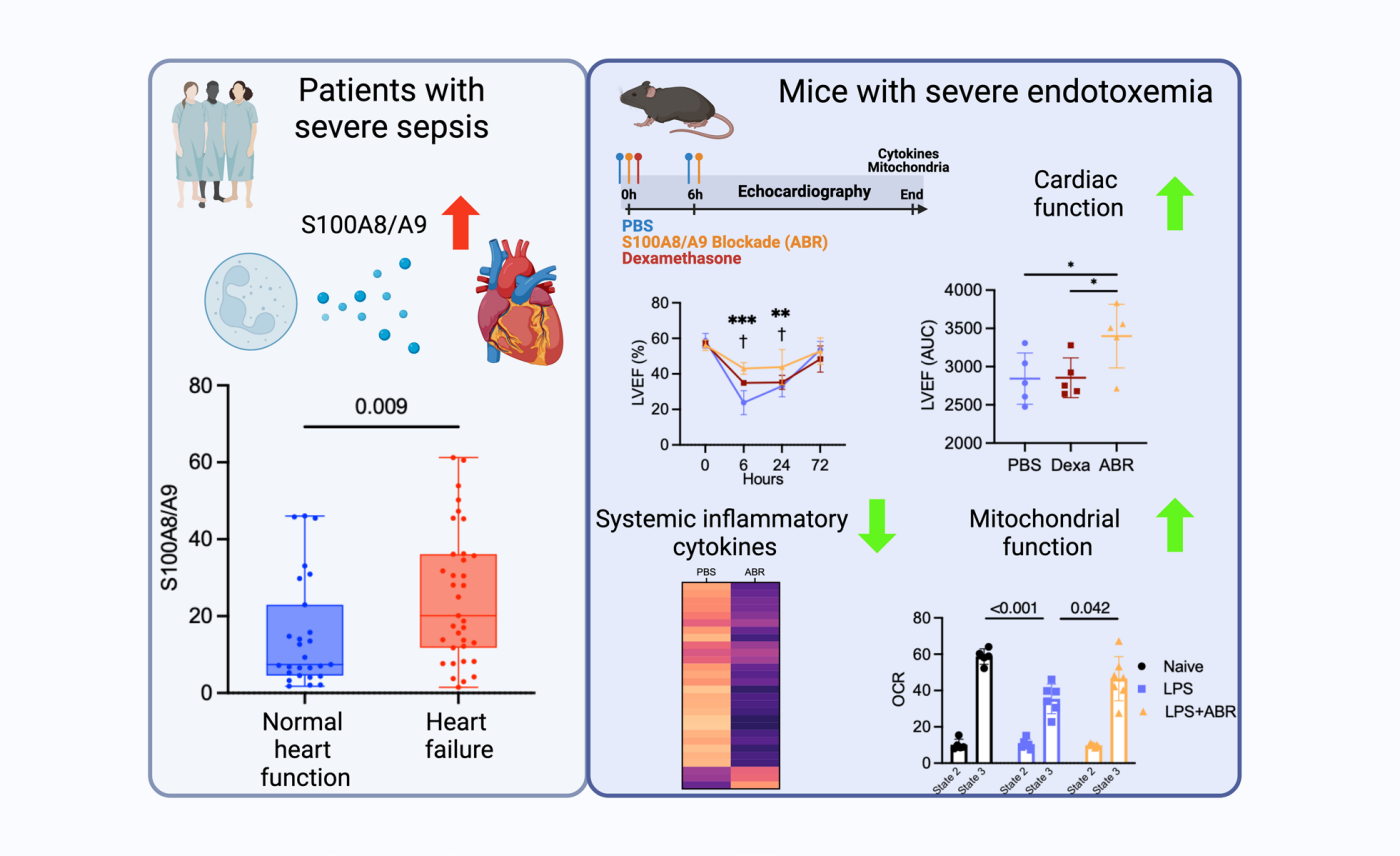

**Supplementary Information:**

The online version contains supplementary material available at 10.1186/s13054-023-04652-x.

## Introduction

Severe sepsis leads to life-threatening multi-organ dysfunction caused by a dysregulated immune and inflammatory response to infection [1, 2]. In the United States alone, sepsis accounts for around 10% of all ICU admissions, with the number of cases exceeding 750,000 per year [2]. Sepsis is associated with a 20–30% mortality rate and is responsible for over 200,000 deaths annually in the United States. The most common causes for sepsis are pneumonia, intraabdominal infection, and urinary tract infection [2]. The central cause of mortality in sepsis patients is cardiovascular collapse, leading to multiple organ failure [3]. Vascular leakiness, causing massive loss of fluid into the tissues, and a marked decrease in vascular tonus lead to hypotension and impaired peripheral perfusion. Consequently, fluid resuscitation and vasopressors are first-line treatments in septic patients [1]. A large proportion of patients with severe sepsis also develop impaired myocardial contractility. This phenomenon, known as sepsis-induced myocardial dysfunction (SIMD), was first described by Calvin et al. [4]. SIMD is generally defined as an acute and reversible global cardiac dysfunction with reduced contractility, often associated with left ventricular (LV) dilation, diminished response to fluid resuscitation and catecholamines, and the absence of acute coronary syndrome as etiology [5]. Echocardiographically, there is high heterogeneity among sepsis patients, including subgroups with normal, depressed or elevated LV ejection fraction (LVEF) or LV volumes [6]. Depressed myocardial strain was found to be significantly associated with increased mortality in sepsis patients, suggesting that the presence of SICM might contribute to a poor prognosis [7]. Consensus has yet to be reached regarding best practice for echocardiographic evaluation for diagnosis and prognosis in sepsis. The mechanisms behind cardiac dysfunction in sepsis include impaired myocardial circulation, mitochondrial dysfunction, downregulation of beta adrenoreceptors and direct myocardial depressants acting through receptors present on cardiomyocytes [8–10]. These mechanisms are not well understood and there are currently no available therapies able to reverse myocardial dysfunction in severe sepsis [11]. Inflammatory cytokines such as IL-1β and TNF-α have been proposed to be involved in SIDM but randomized control trials targeting either cytokine in sepsis patients failed to improve cardiac function or reduce mortality [12].

S100A8 and S100A9 are EF-hand proteins that form the S100A8/A9 heterodimer, known as calprotectin or MRP-8/14. S100A8/A9 is a potent pro-inflammatory alarmin stored in large amounts in neutrophils and released upon activation [13]. Neutrophils are the first immune responders to bacterial challenge, followed by monocytes and other innate and adaptive immune cell populations [14, 15]. S100A8/A9 signals mainly through the pattern recognition receptors (PRR) Toll-like receptor 4 (TLR-4) and the Receptor for Advanced Glycation End-products (RAGE), and has activating effects on a wide range of immune and non-immune cells, as well as chemotactic effects on neutrophils [16–18]. In clinical studies, plasma S100A8/A9 was found to be elevated in sepsis patients and to correlate with increased mortality and poor prognosis [19–25]. Likewise, mice deficient in S100A8/A9 are protected against the development of endotoxemia-induced mortality [26]. Taken together, these data indicate that S100A8/A9 might have a causal effect in the development of severe sepsis and might be therapeutically targeted to ameliorate disease severity.

In the present work, we investigated the relationship between systemic S100A8/A9 release and myocardial dysfunction in a cohort of patients with severe sepsis treated in the intensive care unit (ICU). Further, we explored whether S100A8/A9 blockade with a small molecule inhibitor could be used as a potential treatment against systemic inflammation and acute myocardial dysfunction in an experimental model of endotoxemia.

## Methods

### Clinical study population

S100A8/A9 was measured in plasma from 62 sepsis patients included in the Sepsis in the Intensive Care Unit-2 (SICU-2) study (NCT04695119), a prospective observational study with the overall aim to explore the associations between biomarkers, hemodynamic and echocardiographic measurements, and clinical outcomes in patients with septic shock. Study subjects were patients ≥ 18 years of age admitted to the ICU of Linköping University Hospital, Sweden, and diagnosed with septic shock according to the Sepsis III definition [27]. The subjects were included between September 2018 and November 2021. All patients were included in the study within 12 h from ICU admission and gave written informed consent. The study was approved by the regional ethics committee for human research in Jönköping, Sweden and by the Swedish ethical review authority, and was conducted according to the ethical guidelines of the Declaration of Helsinki.

### Patient echocardiography

Transthoracic echocardiography was performed within 24 h of study inclusion. A GE Vingmed Ultrasound Vivid E95 or Vivid S70 echocardiography scanner was used for data acquisition. A comprehensive echocardiogram was performed according to a prespecified study protocol by experienced sonographers or a clinical physiologist. Images were transferred to ViewPoint (v. 6.10.1, GE Healthcare GmbH, Solingen, Germany) with EchoPAC Suite (GE EchoPAC PC Software only v. 202, GE Vingmed Ultrasound AS, Horten, Norway). At least three heart beats were recorded in each view for patients with sinus rhythm, and at least five beats in patients with atrial fibrillation. Two or more of the following criteria were used to define the presence of left ventricular dysfunction: left ventricular ejection fraction (LVEF) ≤ 50%, average mitral (lateral and medial) s’ measured by color tissue Doppler < 5 cm/s, average of lateral and medial mitral annular plane systolic excursion (MAPSE) < 10 mm or GLS (average of apical 4-chamber and 2-chamber views) > − 15% [28]. Currently, there are no clear guidelines for echocardiographic evaluation of cardiac function in sepsis. Non-cardiac factors such as reduced afterload related to vasoplegia have a strong influence on LVEF and LV volumes, leading to high heterogeneity among sepsis patients [6]. LVEF alone is considered to have a poor diagnostic and prognostic value in SIMD [5, 6]. We followed the American Society for Echocardiography recommendations for quantification of LV systolic function [29] and the PRICES consensus recommendations for reporting critical care echocardiography research studies. These guidelines recommend evaluation of MAPSE, s’, and GLS together with LVEF to provide additional information in the context of critical illness [29, 30]. We defined the presence of LV dysfunction based on abnormality of two of the four proposed criteria in an attempt to increase diagnostic accuracy.

### Biochemical analysis

Blood was collected in BD Vacutainer® EDTA tubes (Beckton Dickinson), centrifuged at 2000 g for 10 min, and plasma was aliquoted and stored at − 80 °C before analysis. S100A8/A9 was measured using an Enzyme Linked Immunosorbent Assay (ELISA) according to manufacturer’s instructions (BMA Biomedicals, Switzerland). Inter assay variability was 8.5%.

### Animal models and in-vivo treatments

C57Bl/6NrJ mice were purchased from Janvier Labs. Females aged 8–12 weeks were used for all experiments. S100A9^−/−^ mice on C57Bl/6 background were generously provided by Prof. Thomas Vogl (Muenster University, Germany) and bred in-house. Endotoxemia was induced by intraperitoneal injection of 5 mg/kg lipopolysaccharide (LPS) in female mice. We elected not to use male mice in these experiments, as they develop a more severe inflammatory response to LPS [31], leading to high mortality during the first 24 h. Early S100A8/A9 blockade was achieved by intraperitoneal administration of 30 mg/kg of the small-molecule inhibitor ABR-238901 at 0 and 6 h. ABR-238901 binds directly to S100A9 and S100A8/A9 and inhibits the interaction of the proteins with their cognate receptors [32]. For the experiments exploring the effects of delayed treatment, ABR-238901 was administered at 12 and 18 h post-LPS and the experiments terminated at 24 h. In separate experiments, 2 mg/kg dexamethasone was administered intraperitoneally directly after LPS, according to previously published experimental protocols [33], to compare the effects of S100A8/A9 blockade with corticosteroid treatment. Mice were randomly assigned to experimental groups and experiments were carried out in a blinded fashion. All experimental procedures were approved by the regional ethics committee in Lund and were performed in compliance with the EU guidelines.

### Animal echocardiography

Depending on the experiment, ultrasound measurements were performed at 0 (baseline), 6, 12, 24 and 72 h post-LPS injection, as specified in Results. Transthoracic echocardiography was performed using the Vevo 3100 imaging system (VisualSonics) using a MX-550D probe. Anesthesia was induced with 4% isoflurane and maintained with 2% isoflurane. The animals were placed in the supine position, shaved with depilatory cream and pre-warmed ultrasound gel was applied to the chest. The mice were maintained at 37 °C during the measurements. Images were collected in the parasternal long axis in the center of the heart with apex and aorta as reference, in accordance with the small-animal echocardiography guidelines [34]. Left ventricular function was analyzed by Vevo LAB (VisualSonics) from B-mode images by tracing the left ventricle in systole and diastole.

### Cardiac flow cytometry

Mice were sacrificed by isoflurane overdose followed by cervical dislocation. Residual cardiac blood was evacuated by left ventricular perfusion with 20 mL PBS before cardiac isolation. Both atria were removed, the hearts were finely minced with surgical scissors and placed into tubes with 1 mL digestion mix containing Collagenase I, 450 U/mL; Collagenase XI, 125 U/mL; DNAase I, 60 U/mL; Hyaluronidase, 60 U/mL purchased from Sigma-Aldrich in Ca^2+^ and Mg^2+^-free PBS. The tubes were incubated for 45 min at 37 °C, 450 RPM shaking, and single-cell suspensions were obtained by passing through 70 µm cell strainers, followed by washing and staining. Unspecific binding was blocked by incubation with an anti-CD16/32-antibody for 5 min on ice. Cells were stained for viability using Fixable Live/Dead Zombie/Aqua (Invitrogen) for 10 min on ice, followed by staining with fluorochrome-conjugated antibodies in PBS supplemented with 0.5% BSA and 2.5 mM EDTA for 30 min on ice. The following antibodies were used for flow cytometry analysis: Ly-6G (1A8, Biolegend), CD45.2 (30F-11, Biolegend), Siglec-F (E50-2440, BD Biosciences), CD11b (M1/70, Biolegend), CD64 (X54-5/7.1, Biolegend), MHC-II (M5/114.15.2, Biolegend), Ly-6C (HK1.4, Biolegend), TCRb (H57-597, Biolegend). Samples were analyzed on a Gallios Flow cytometer (Beckman Coulter) and data analysis was carried out using FlowJo (BD Biosciences).

### Quantitative PCR

Murine cardiac tissue was homogenized in TRI Reagent® (Thermo Scientific) using a tissue rotor–stator. Total RNA was isolated according to manufacturer’s instructions. cDNA was generated from 1 µg RNA using High-Capacity RNA to cDNA Kit (Applied Biosystems). Quantitative real-time PCR was performed using TaqMan Fast Mastermix (Applied Biosystems) using the following TaqMan primers (Applied Biosystems): *Nlrp3 (Mm00840904_m1), Casp-1 (Mm00438023_m1), Il-1b (Mm00434228_m1), Il-18 (Mm00434225_m1), Tnf-α (Mm00443258_m1), Il-6 (Mm00439653_m1), S100a8 (Mm00496696_g1), S100a9 (Mm00656925_m1), Bnp (Mm01255770_g1), Gapdh (Mm99999915_g1).* Quantitative PCR was run on a ViiA7 Real-Time PCR System (Applied Biosystems) and fold change was calculated using the 2^−ΔΔCt^ method.

### Murine plasma analysis

Blood was collected from the left ventricle upon sacrifice into EDTA-coated tubes and plasma was isolated by centrifugation at 1000 g for 10 min and immediately stored at –80 °C until further analysis. For S100A8/A9 kinetics in plasma, saphenous vein blood was collected into EDTA-coated capillary tubes at 0, 6, 12, 24, 48, 72 and 96 h after LPS administration. Plasma levels of S100A8/A9 were measured by DuoSet® ELISA (R&D Systems, DY8596-05), according to the manufacturer’s instructions. All samples were diluted 1:100 prior to analysis. Color was developed using 1-Step™ Ultra-TMB-ELISA (Thermo Scientific). Murine cytokines were measured by multiplex-bead assay using the 36-Plex Mouse ProcartaPlex™ Panel 1A (Invitrogen) according to manufacturer’s instructions and analyzed in a flow-based Bio-Plex 200 system (Bio-Rad). For heatmap representation of cytokine data, Z-scores were calculated, clustered using Clustergrammar and graphed using GraphPad Prism 9.

### Analysis of mitochondrial respiration and ROS production in vitro

Mouse subsarcolemmal (SSM) and interfibrillar (IFM) mitochondria were isolated from whole hearts by differential centrifugation at 24 h post-LPS, as previously described [35, 36]. Mitochondrial respiration was assessed using a Clark-type electrode (Hansatech, UK) at room temperature after the addition of the substrates of complex I (malate and glutamate) or complex II (succinate and rotenone) to assess basal respiration (state 2). Maximal oxygen consumption (state 3) was activated by the addition of 200 µmol/L ADP. Oxygen consumption was expressed as nmols O2/min*CS. Hydrogen peroxide (H_2_O_2_) production was assessed from changes in fluorescence after the addition of respiration substrates in the presence of Amplex® Red (10 µmol/L) and horseradish peroxidase (5 U/mL). Changes in fluorescence over time were monitored at 590 nm using a multimode reader (iD3 SpectraMax, Molecular devices). H_2_O_2_ concentration was calculated using standard H_2_O_2_ curves and rates were expressed as pmol H_2_O_2_/min/mg protein.

### Statistical analysis

For analysis of patient data, Mann–Whitney test was used for continuous variables and Chi-squared test for categorical variables. A receiver operating curve (ROC) was constructed for the relationship between S100A8/A9 levels and the presence of left-ventricular dysfunction in sepsis patients. The correlation between S100A8/A9 and the sequential organ failure assessment (SOFA) score at admission was assessed by the Spearman rank test. For longitudinal experiments, repeated measures 2-way ANOVA was employed and significance at individual time points was calculated with Fischer’s LSD test. Statistical differences between two groups were examined with the Student’s *t*-test or Mann–Whitney test, depending on data normality. Data distribution was assessed with the Shapiro–Wilk test. The statistical differences among three groups were assessed by 1-way ANOVA and Fisher’s LSD Test for normally distributed data or by the Kruskal–Wallis test for non-normally distributed data. Statistical analysis was carried out using GraphPad Prism 9.0. All data are presented as mean ± standard deviation (SD).

## Results

### S100A8/A9 is elevated in severe sepsis patients with left ventricular dysfunction

**Fig. 1:**
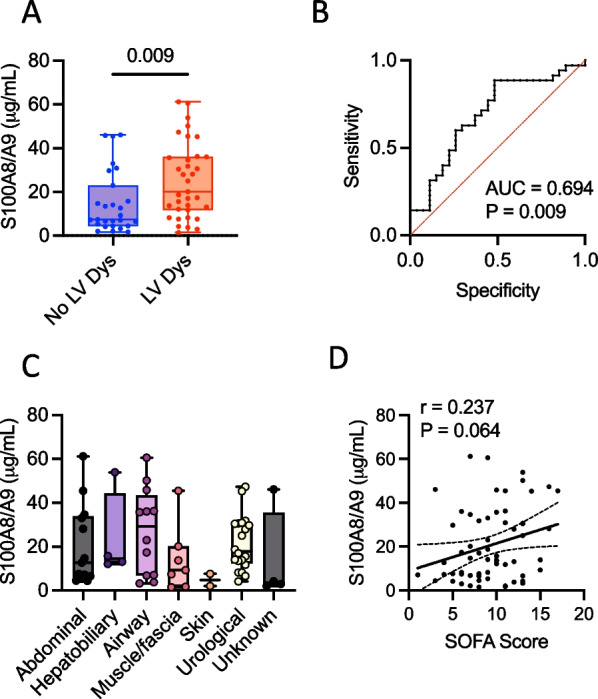
S100A8/A9 is elevated in critically ill sepsis patients with left ventricular dysfunction. S100A8/A9 was measured in plasma collected upon admittance into the ICU. **A** Comparison of plasma S100A8/A9 levels in patients with severe sepsis, with or without left ventricular dysfunction (LV Dys). **B** ROC analysis of the ability of S100A8/A9 at admission to identify LV Dys. **C** Relationship between S100A8/A9 levels and the site of infection. **D** Correlation between plasma S100A8/A9 and SOFA disease severity score. Comparison of S100A8/A9 in patients with or without LV Dys was performed using the Mann–Whitney U test. Spearman correlation analysis was used to assess the relationship between S100A8/A9 and the SOFA score

A total of 62 consecutive patients with severe sepsis admitted to the ICU at Linköping University Hospital, Sweden during 2018–2021 were included into the study. The presence of left ventricular (LV) dysfunction was determined by echocardiography at ICU admission as described in Methods. We found a total of 35 patients (56%) with LV dysfunction. The patients with LV dysfunction had significantly higher disease severity compared to patients with normal LV function, as measured by the SOFA (9 vs. 10, *P* = 0.047) and SAPS (59 vs. 66, *P* = 0.029) clinical scores (Table 1). Plasma S100A8/A9 was also significantly higher in patients with LV dysfunction (20.1 μg/mL vs. 7.4 μg/mL, *P* = 0.009, Table 1 and Fig. 1A). The ROC analysis demonstrated that plasma S100A8/A9 levels are able to identify sepsis patients with LV dysfunction (Fig. 1B, c-statistic = 0.694, *P* = 0.009). The levels of plasma S100A8/A9 were not dependent on the site of infection (Fig. 1C). We also found a modest correlation between S100A8/A9 and disease severity, assessed by the SOFA score (Fig. 1D r = 0.237, *P* = 0.064).

**Table 1:** Clinical characteristics of the study cohort

Demographics	Total (62)	No left ventricular dysfunction (27)	Left ventricular dysfunction (35)	*P*-value
Age	72 (59–76)	65 (56–75)	73 (62–76)	0.119
Sex, male n (%)	30 (48.2)	13 (48.15)	17 (48.6)	0.974
SAPS	64 (56–75)	59 (52–69)	66 (58–76)	0.029
SOFA	9 (7–12)	9 (5–11)	10 (8–13)	0.047
Clinical frailty scale	3 (2–4)	2 (2–4)	3 (2–4)	0.241
Coronary artery disease, n (%)	9 (14.5)	3 (11.1)	6 (17.1)	0.504
Heart failure, n (%)	9 (14.5)	2 (7.4)	7 (20.0)	0.163
Diabetes mellitus, n (%)	10 (16.1)	4 (14.8)	6 (17.1)	0.805
Stroke or TIA, n (%)	10 (16.1)	3 (11.1)	7 (20.0)	0.345
Hypertension, n (%)	43 (69.4)	15 (55.6)	28 (80.0)	0.038
S100A8/A9 (µg/mL)	14.3 (6.9–32.1)	7.4 (4.5–22.9)	20.1 (11.8–36.1)	0.009

The values are presented as median (IQR) or percentages. Statistical testing for continuous variables was performed with the Mann–Whitney U test, and for categorical variables with the Chi-squared test. SAPS, Simplified Acute Physiology Score; SOFA, Sequential Organ Failure Assessment; TIA, Transient ischemic attack

### S100A8/A9 blockade improves myocardial dysfunction during endotoxemia

**Fig. 2:**
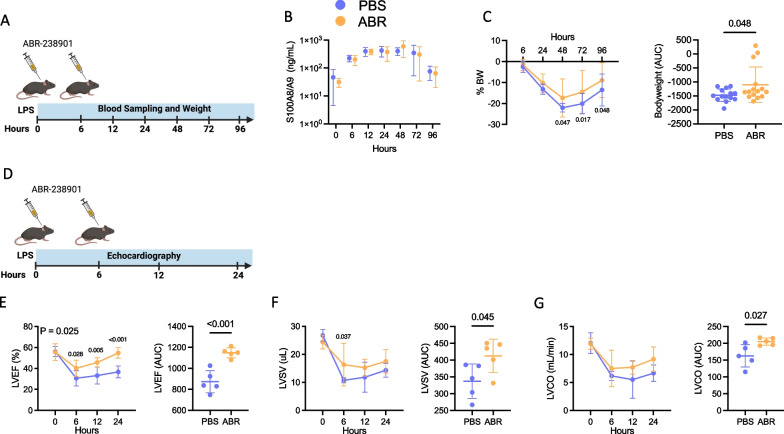
S100A8/A9 blockade ameliorates septic cardiomyopathy. Endotoxemia was induced in C57Bl/6NrJ mice by intraperitoneal injection of 5 mg/kg LPS. The mice were treated with 30 mg/kg ABR-238901 (ABR) or PBS intraperitoneally at 0 h and 6 h post-LPS. **A** Experimental timeline for **B**–**C**. Blood sampling was performed at 0, 6, 12, 24, 48, 72 and 96 h post-LPS. **B** Kinetics of S100A8/A9 release. **C** Bodyweight change, expressed as serial measurements and area under the curve (AUC). **D** Experimental timeline for **E**–**I**. Echocardiography was performed at baseline, 6 h, 12 h and 24 h. **E**–**G** Left ventricular ejection fraction (LVEF), stroke volume (LVSV) and cardiac output (LVCO), presented as serial measurements and area under the curve over time. Statistical testing was performed by repeated-measures two-way ANOVA with Fisher’s LSD Test. The p-values on the time-course graphs reflect differences between the treatment groups at the respective time point. Differences in AUC between the groups were assessed using Student’s *t*-test, following normality assessment with Shapiro–Wilk test. PBS, Phosphate Buffered Saline; ABR, ABR-238901; BW, bodyweight; AUC, area under curve; LVEF, Left ventricular ejection fraction; LVSV, Left ventricular stroke volume; LVCO, Left ventricular cardiac output. Data is represented as mean ± SD. **B** and **C** N = 7–15 per group, **E**–**G** N = 5 per group

We used an experimental model of endotoxemia to investigate whether S100A8/A9 has a direct pathogenic role and may be used as a therapeutic target in SIMD (Fig. 2A). Plasma S100A8/A9 levels were elevated as early as 6 h and remained stably increased for 72 h after administration of a single 5 mg/mL LPS injection (Fig. 2B). S100A8/A9 blockade was induced by intraperitoneal administration of the small-molecule inhibitor ABR-238901, which binds directly to S100A9 and S100A8/A9 and inhibits the interaction with their cognate receptors [32]. S100A8/A9 blockade with ABR-238901 did not impact S100A8/A9 levels (Fig. 2B). Following induction of endotoxemia, the mice experienced a rapid loss in bodyweight, which was partially reversed by S100A8/A9 blockade (Fig. 2C). To examine the effects of S100A8/A9 blockade on cardiac function, we performed echocardiography at baseline, 6, 12 and 24 h post-LPS (Fig. 2D). LV systolic function was significantly improved by the treatment over the course of the experiment, as assessed by repeated measures 2-way ANOVA (RM-ANOVA, *P* = 0.025) and area under-the-curve (AUC) comparison (*P* < 0.001). The post-hoc analysis revealed significantly higher LVEF at 6 h (40.2% vs. 30.7%, *P* = 0.028), 12 h (45.7% vs. 33.2%, *P* = 0.005) and 24 h (54.7% vs. 36.7%, *P* < 0.001) in mice receiving S100A8/A9 blockade (Fig. 2E). LV stroke volume and LV cardiac output were also significantly higher in treated mice (Fig. 2F and G). Taken together, these results indicate that S100A8/A9 has a direct pathogenic role in systolic LV dysfunction during endotoxemia, which can be reversed by the ABR-238901 treatment.

### S100A8/A9 blockade dampens systemic and myocardial inflammation

**Fig. 3:**
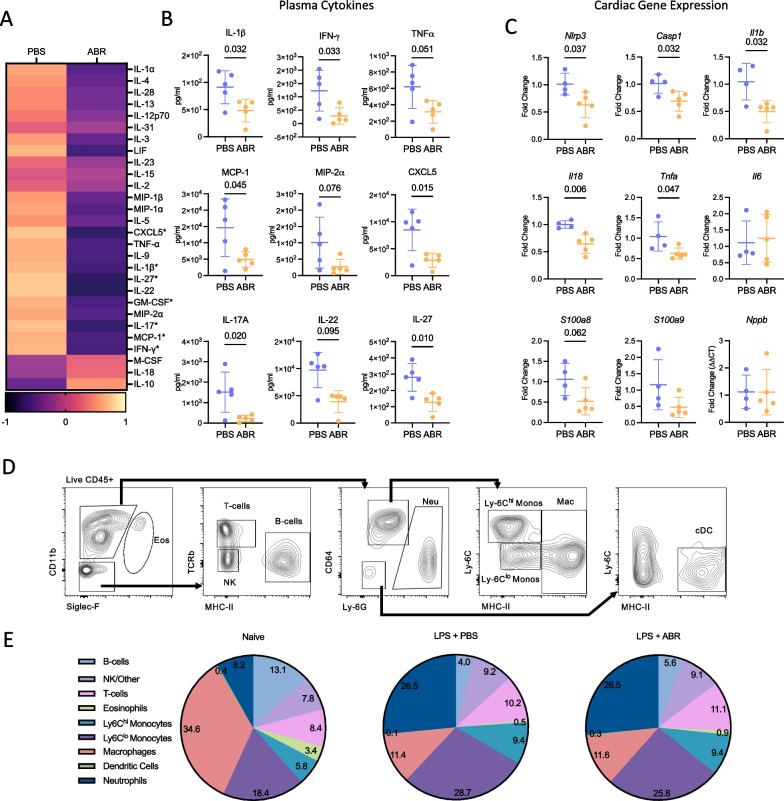
S100A8/A9 blockade inhibits systemic inflammation but does not impact cardiac immune cell environment during endotoxemia. Endotoxemia was induced in C57Bl/6NrJ mice by intraperitoneal injection of 5 mg/kg LPS. The mice were treated with 30 mg/kg ABR-238901 or PBS intraperitoneally at 0 h and 6 h post-LPS. The mice were sacrificed at 24 h for plasma isolation and flow cytometric analysis (**A**, **B**, **D**, **E**), or at 12 h for cardiac gene expression (**C**). Cytokine levels were measured in plasma. **A** Heatmap of plasma cytokine changes following ABR-238901 treatment during endotoxemia, expressed as Z-score in relation to median. **B** Treatment effect on plasma levels of individual pro-inflammatory cytokines and chemokines. **C** Cardiac gene expression of NLRP3 inflammasome components, inflammatory cytokines, S100A8, S100A9 and BNP. **D** Gating strategy for flow cytometry analysis of cardiac CD45+ leukocytes. **E** Distribution of cardiac immune cell populations during endotoxemia, with and without S100A8/A9 blockade. Statistical differences between two groups were examined with Student’s *t*-test or Mann–Whitney test, following normality assessment with Shapiro–Wilk test. *P < 0.05, PBS, Phosphate Buffered Saline; ABR, ABR-238901; Eos, Eosinophils; NK, Natural Killer cells; Neu, Neutrophils; Monos, Monocytes; Mac, Macrophages; cDC, conventional dendritic cells. Data is represented as mean ± SD. **A** and **B** N = 5 per group, **C** N = 4-5 per group, **D** and **E** N = 5–8 per group

To investigate the anti-inflammatory effects of S100A8/A9 blockade, plasma was collected at 24 h and cytokine levels were evaluated using a multiplex bead assay. Inhibition of S100A8/A9 signaling resulted in a broad reduction of systemic inflammatory cytokine levels (Fig. 3A). S100A8/A9 blockade reduced the inflammatory cytokines IL-1β, IFN-γ, and TNF-α, as well as the myeloid mediators MCP-1, MIP-2α and CXCL5 (Fig. 3A, B). The Th17 T cell cytokines IL-17A and IL-22, involved in neutrophil recruitment [37], as well as the immunomodulatory cytokine IL-27 were also lowered by the treatment (Fig. 3A, and B) [38]. To investigate the effects of ABR-238901 on myocardial inflammation, we performed qPCR analysis of cardiac tissue 12 h post-LPS (Fig. 3C). The treatment significantly reduced cardiac expression of TNF-α and of the inflammasome components NLRP3, caspase-1, IL-1β and IL-18. There was also a tendency towards reduced cardiac mRNA expression of S100A8 and S100A9 (Fig. 3C).

We also investigated whether the treatment impacts cardiac immune cell infiltration. Hearts from naïve and endotoxemic mice with and without treatment were isolated at 24 h post-LPS and analyzed by flow cytometry (Fig. 3D and E). We found important changes in the distribution of CD45^+^ immune cells between naïve and endotoxemic mice, characterized by a larger proportion of neutrophils and monocytes, and a reduced proportion of macrophages, eosinophils, and B cells (Fig. 3E). Cardiac immune cell infiltration was unaffected by S100A8/A9 blockade, suggesting that the observed beneficial effects of the treatment are not mediated by modulation of the immune cell recruitment into the heart (Fig. 3E, Additional file 1: Fig. 1).

### S100A8/A9-deficient mice are protected against LPS-induced cardiac dysfunction and systemic inflammation

**Fig. 4:**
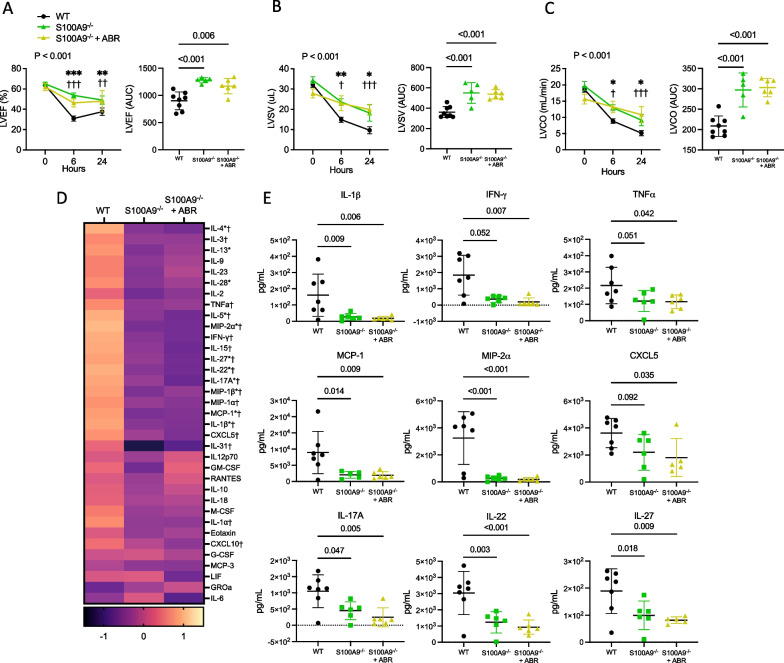
S100A9-deficient mice are partially protected against systemic inflammation and left ventricular dysfunction during endotoxemia. Endotoxemia was induced in wild-type or S100A9^−/−^ C57Bl/6NrJ mice by intraperitoneal injection of 5 mg/kg LPS. In the ABR-238901-treated group, the S100A9^−/−^ mice received 30 mg/kg ABR-238901 intraperitoneally at 0h and 6h post-LPS. Echocardiography was performed at baseline, 6 h and 24 h. Mice were sacrificed at 24 h for plasma collection and cytokines were measured in plasma using multiplex bead assay. **A**–**C** Left ventricular ejection fraction, stroke volume and cardiac output presented as serial measurements over time, with the corresponding area under the curve (AUC). **D** Heatmap of changes in plasma cytokines in wild-type and S100A9^−/−^ mice with and without ABR-238901 treatment, expressed as Z-scores. **E** Individual plasma cytokine and chemokine levels. Statistical testing of echocardiographic parameters over time was performed using repeated-measures 2-way ANOVA with Fisher’s LSD Test. *P*-values reflect differences between treatment groups over time. Symbols reflect the difference between the treatment groups at the respective time point. Statistical differences between 3 groups were assessed with 1-way ANOVA with Fisher’s LSD Test or Kruskal–Wallis test, following normality assessment with Shapiro–Wilk test. *, S100A9^−/−^ versus WT; *P < 0.05, **P < 0.01, ***P < 0.001; †, S100A9^−/−^ + ABR versus WT; † *P* < 0.05, †† *P* < 0.01, ††† *P* < 0.001; WT, wild-type; ABR, ABR-238901; AUC, area under curve; LVEF, Left ventricular ejection fraction; LVSV, Left ventricular stroke volume; LVCO, Left ventricular cardiac output. Data is represented as mean ± SD. N = 5–8 per group

To confirm the pathogenic role of S100A8/A9 in SIMD, we used S100A9-deficient mice that also lack S100A8 and the S100A8/A9 heterodimer [39]. Echocardiography was performed in endotoxemic wild-type mice, S100A9^−/−^ mice and S100A9^−/−^ mice treated with ABR-238901. The S100A9^−/−^ mice were partially protected against disease development compared to wild type mice, with a significantly higher LVEF at 6 h post-LPS (53.7% vs. 30.9%, *P* < 0.001). S100A8/A9 inhibition with ABR-238901 did not induce further LVEF improvement, confirming the specificity of the blocker (Fig. 4A). LVEF remained higher in the two S100A9^−/−^ groups at 24 h post LPS (*P* < 0.01 vs. wild-type). There was a significant difference between the systolic LV function in the wild-type controls compared to the S100A9^−/−^ groups across the duration of the experiment, as assessed by repeated measures 2-way ANOVA (*P* = 0.001) and AUC comparison (*P* < 0.001). Mice lacking S100A8/A9 also displayed higher stroke volume (*P* < 0.001) and cardiac output (*P* < 0.001) compared to wild type animals (Fig. 4B and C). Blockade of S100A8/A9 in S100A9^−/−^ mice did not lead to any additive effects on LV functional or structural parameters compared to PBS-treated S100A9^−/−^ controls.

Similar to wild-type mice receiving S100A8/A9 blockade, S100A9^−/−^ mice had a highly reduced systemic inflammatory response to LPS (Fig. 4D and E). All inflammatory mediators that were downregulated by the ABR-238901 treatment (Fig. 3A and B) were also decreased in LPS-treated S100A9^−/−^ mice (Fig. 4D and E). We also found significantly lower levels of IL-4, IL-5, IL-13, IL-28, and MIP-1b (Fig. 4D), further highlighting the central role of S100A8/A9 in mounting a broad immune response to LPS. Taken together, these results confirm the pro-inflammatory and cardio-depressant role of S100A8/A9 in endotoxemia and confirm the target specificity of the ABR-238901 treatment.

### Delayed S100A8/A9 blockade reverses established systolic LV dysfunction

**Fig. 5:**
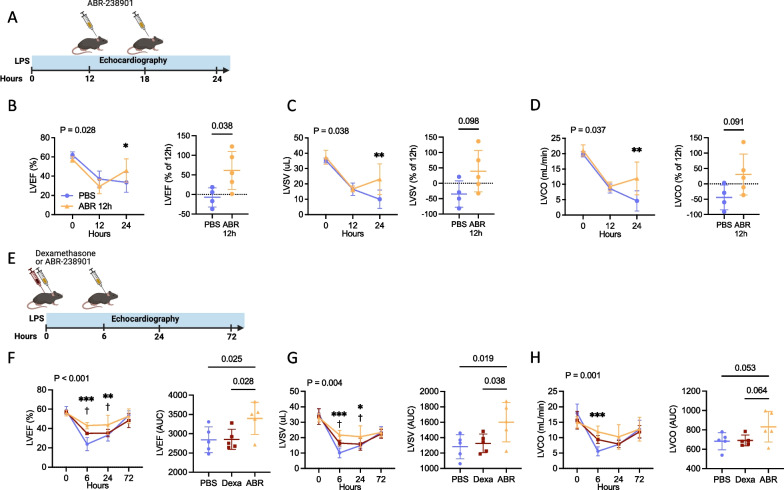
ABR-238901 reverses established cardiac dysfunction and is a more efficient treatment compared to Dexamethasone. **A**–**E** Delayed ABR-238901 treatment starting from 12 h post LPS successfully rescues cardiac dysfunction during endotoxemia. **A** Experimental layout for **B**–**D**. **B** Repeated LVEF measurements and % LVEF change from treatment start to 24h post-LPS. **C** Absolute LVSV and % LVSV change from treatment start. **D** Absolute LVCO and % LVCO change from treatment start. **E** Experimental layout for **F**–**H**. **F**–**H** Comparison between treatment with ABR-238901 and Dexamethasone on cardiac function during endotoxemia. Following disease induction, the mice were treated either with 2 mg/kg Dexamethasone at 0h or with ABR-238901 at 0 and 6 h post-LPS. **F**–**H** LVEF, LVSV and LVCO over time and area under the curve. Statistical testing of echocardiographic parameters over time was performed by repeated-measures 2-way ANOVA with Fisher’s LSD Test. The *P*-values reflect the difference between treatment groups over time. The symbols reflect the difference between treatment groups at the respective time point. The statistical difference between two groups was tested with Student’s *t*-test. Differences between three groups were tested using 1-way ANOVA with Fisher’s LSD Test. Normality assessment was performed with Shapiro–Wilk test. *, ABR versus PBS; *P < 0.05, **P < 0.01, ***P < 0.001; †, ABR vs Dexa; † *P* < 0.05; PBS, Phosphate Buffered Saline; ABR, ABR-238901; Dexa, Dexamethasone; AUC, area under curve; LVEF, Left ventricular ejection fraction; LVSV, Left ventricular stroke volume; LVCO, Left ventricular cardiac output. Data is presented as mean ± SD. N = 4–5 per group

In the clinical setting, sepsis patients are generally admitted with already established disease. To replicate this scenario, we initiated the ABR-238901 treatment at 12 h post-LPS and assessed cardiac function by serial echocardiography (Fig. 5A). The late treatment initiation also avoids potential interference by the S100A8/A9 blocker with the initial response to LPS. LV systolic function was highly depressed in both groups at the start of the treatment, with no inter-group difference (Fig. 5B–D). Delayed S100A8/A9 blockade efficiently rescued LVEF at 24 h (45.9% vs. 33.6%, *P* = 0.036), leading to a 61% relative LVEF increase from 12 to 24 h compared to a 7.5% relative decrease in PBS-treated controls (Fig. 5B, *P* = 0.040). Repeated measures 2-way ANOVA revealed a significant difference between the treatment groups over time for LVEF (*P* = 0.028), stroke volume (*P* = 0.038) and cardiac output (*P* = 0.037) (Fig. 5B–D).

### ABR-238901 outperforms dexamethasone in preventing cardiac dysfunction

Patients with severe sepsis, cardiovascular collapse and multiorgan failure that do not respond to conventional treatment are sometimes treated with glucocorticoids, in an effort to reduce the potent systemic inflammation driving disease pathogenesis. To assess S100A8/A9 blockade as a viable treatment alternative, we compared the effects of Dexamethasone and ABR-238901 on cardiac dysfunction during endotoxemia (Fig. 5E). Administration of Dexamethasone immediately after LPS improved LVEF at 6 h post-LPS (34.9% vs. 23.8%, *P* = 0.003) compared to controls, but there was no difference between the groups over the three-day course of the experiment, as assessed by LVEF AUC comparison (*P* = 0.957, Fig. 5F). In contrast, ABR-238901 significantly improved LVEF at 6 h post-LPS compared to controls (43.0% vs. 23.8%, *P* < 0.001) and also compared to mice receiving Dexamethasone (43.0% vs. 34.9%, *P* = 0.026). Improved LVEF was also seen at 24 h compared to controls (43.8% vs. 35.2%, *P* = 0.004) and Dexamethasone (43.8% vs. 35.2%, *P* = 0.018), as well as over the entire follow-up period, as assessed by repeated measures 2-way ANOVA (*P* < 0.001) and AUC comparison (*P* = 0.0.25 vs. wild-type; *P* = 0.028 vs. Dexamethasone). The LVEF increase was associated with significant improvements in LV stroke volume and cardiac output, compared to both PBS- and Dexamethasone-treated groups (Fig. 5G and H).

### S100A8/A9 blockade ameliorates cardiac mitochondrial dysfunction in endotoxemia

**Fig. 6:**
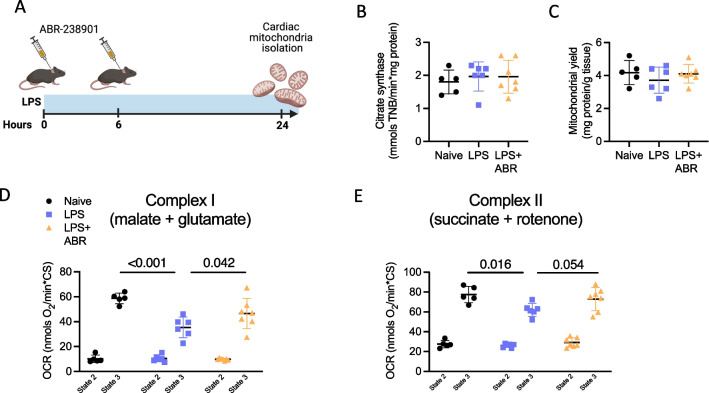
S100A8/A9 blockade improves LPS-induced mitochondrial dysfunction in the heart. **A** Experimental layout. **B** Citrate synthase activity. **C** Mitochondrial yield. **D** Oxygen consumption rate (OCR) after incubation with respiratory substrates (malate + glutamate) feeding complex I, either in the absence (state 2) or presence (state 3) of ADP. **E** Oxygen consumption rate after incubation with respiratory substrates (succinate + rotenone) feeding complex II. Differences between three groups were tested using 1-way ANOVA with Fisher’s LSD Test. Normality assessment was performed with Shapiro–Wilk test. LPS, Lipopolysaccharide; ABR, ABR-238901; OCR, Oxygen consumption rate. Data are represented as mean ± SD from N = 4–7 per group

Mitochondrial dysfunction is thought to play a central role in the pathogenesis of SIMD [10]. To elucidate whether the cardioprotective effect of S100A8/A9 blockade during endotoxemia can be related to preserved mitochondrial function in the heart, we isolated subsarcolemmal mitochondria (SSM) and interfibrillar mitochondria (IFM) from cardiac tissue at 24 h post-LPS (Fig. 6A). No differences were observed in terms of mitochondrial yield or citrate synthase (CS) activity, indicating similar numbers of mitochondria in all groups (Fig. 6B and C, Additional file 2: Fig. 2B, C). Endotoxemia induced a significant decrease in ADP-stimulated (state 3) mitochondrial oxygen consumption in SSM, as compared with mitochondria from control mice, either when incubated with the complex I respiration substrates malate and glutamate (*P* < 0.001, Fig. 6D) or with succinate and rotenone, feeding complex II (*P* = 0.016, Fig. 6E). Mitochondrial basal respiration (state 2) was not affected by endotoxemia. ABR-238901 treatment improved both complex I-mediated (*P* = 0.042) and complex II-mediated (*P* = 0.054) maximal oxygen consumption in SSM. Similar trends in ADP-stimulated respiration were observed in IFM when fed with malate and glutamate (Additional file 2: Fig. 2B, C). Both SSM and IFM from control animals presented a large increase in ROS production in the presence of succinate, as compared with incubation with malate and glutamate (Fig. 6F, Additional file 2: Fig. 2F). This massive ROS production was prevented by the addition of rotenone, indicating that it originates from reverse electron transfer to complex I. The LPS or LPS + ABR-238901 treatments *in-vivo* did not influence succinate-induced ROS production (Additional file 2: Fig. 2F, G).

## Discussion

In the current work, we explored the importance of S100A8/A9 in SIMD and the efficacy of S100A8/A9 blockade with ABR-238901 as a potential treatment in sepsis. We show that elevated plasma S100A8/A9 levels are associated with systolic LV dysfunction in patients with severe sepsis admitted to the ICU. In an animal model of endotoxemia, early or late pharmacologic S100A8/A9 inhibition protected against the development of severe systolic LV dysfunction and reversed the phenotype of already established disease. In a direct comparison experiment, the S100A8/A9 blocker ABR-238901 was more efficient than Dexamethasone in reducing the severity of LV dysfunction. S100A8/A9 blockade prevented the potent systemic inflammatory response induced by endotoxemia. These effects were closely recapitulated by genetic S100A8/A9 deficiency, confirming target specificity. Mechanistically, we show that S100A8/A9 blockade inhibits local inflammation and improves endotoxemia-induced mitochondrial respiratory depression in the myocardium. Our study identifies S100A8/A9 as an important myocardial depressant factor and a viable treatment target in sepsis.

To our knowledge, we are the first to report a link between elevated S100A8/A9 levels and SIMD development in severe sepsis patients requiring ICU care. Previous studies have identified elevated S100A8/A9 levels in patients with sepsis [19, 24, 40] and suggested that high plasma S100A8/A9 might be used as a clinical biomarker to grade disease severity [20]. Additionally, several independent studies have indicated that elevated S100A8/A9 is associated with increased mortality [21–23]. Abnormal LV GLS has also been associated with elevated mortality in sepsis patients [7, 41], suggesting that the development of acute myocardial dysfunction might contribute to the adverse prognosis. The combination of SIMD, vasoplegia and increased vascular permeability leads to cardiovascular collapse, which is the main cause of multi-organ failure and mortality in sepsis patients [3]. Our experimental findings support a causal relationship between S100A8/A9 and the development of myocardial dysfunction. While LPS has a short half-life of a few hours, S100A8/A9 is markedly increased and remains elevated for three days after one dose LPS challenge in mice. Similarly, human subjects injected intravenously with LPS rapidly develop elevated S100A8/A9 levels that remain high for at least 24 h [19]. The systolic LV functional depression in mice follows a similar pattern as plasma S100A8/A9, returning to normal after three days in untreated survivors. The impairment of LV function during this extended period cannot be explained by a direct LPS effect, considering the short half-life of the endotoxin. Although we cannot exclude the parallel influence of other inflammatory mediators, our data demonstrate a direct myocardial depressant role of S100A8/A9 in this context.

Our data support an interdependent relationship between elevated S100A8/A9, systemic inflammatory response and development of cardiac dysfunction in sepsis, and demonstrate that pharmacologic S100A8/A9 blockade reverses the detrimental phenotype [3]. In line with our findings, Vogl et al*.* have previously shown a direct relationship between S100A8/A9 and mortality in an experimental model of endotoxemia [26]. The study reported lower mortality in S100A9^−/−^ mice in response to LPS and *Escherichia coli*-induced abdominal sepsis, which was enhanced by administration of recombinant S100A8/A9. However, the causal relationship between S100A8/A9 and mortality has not been fully elucidated. The authors showed that S100A9-deficient bone marrow cells produce lower levels of the inflammatory cytokines TNF-α and MIP-2 upon LPS stimulation. Here, we report broad downregulation of inflammatory mediators in response to LPS in-vivo, both in S100A9^−/−^ mice and following S100A8/A9 blockade. This is in line with the previously established role of S100A8/A9 as potent upstream activator of the NLRP3 inflammasome and of the NF-kB pathway, through its interaction with TLR-4 and RAGE, leading to production of inflammatory cytokines in various cell types [42–45]. We show that the potent anti-inflammatory effects of S100A8/A9 blockade with ABR-238901 are present both systemically and locally in the myocardium, and likely contribute to the beneficial effects of the treatment on cardiac function. In previous studies using the cecal ligation and puncture (CLP) mouse model of multibacterial abdominal sepsis, we demonstrated that S100A8/A9 binding to TLR-4 and RAGE potently stimulates formation of neutrophil extracellular traps (NETs) and that S100A8/A9 blockade inhibits neutrophil recruitment and lung damage[46, 47]. Together with the findings presented herein, these results show broad anti-inflammatory and organ protective properties of S100A8/A9 blockade in the context of endotoxemia and multibacterial sepsis.

It has previously been demonstrated that cardiac overexpression of S100A8/A9 exacerbates myocardial dysfunction during endotoxemia, supporting a mechanistic link between elevated levels of the protein and impaired contractility [48]. Conversely, ultrasound-guided micro-bubble delivery of S100A9-silencing RNA to cardiomyocytes ameliorated cardiac dysfunction, suggesting that local cardiomyocyte S100A9 expression might, at least partially, mediate the effects [48]. We have also seen a tendency towards reduced cardiac S100A8/A9 mRNA expression in ABR-238901-treated mice. However, this tendency was non-significant and is unlikely to fully explain the beneficial effects of our treatment. In contrast to the above-mentioned study that specifically targeted intracellular S100A8/A9 in cardiomyocytes, we employed systemic extracellular S100A8/A9 blockade with the small molecule inhibitor ABR-238901. ABR-238901 prevents the binding of S100A8/A9 to both TLR-4 and RAGE, leading to a combined effect [32]. Previous studies have identified the expression of both TLR-4 and RAGE in cardiomyocytes [8, 48, 49], and genetic deficiency of TLR-4 in cardiomyocytes is known to protect against cardiac dysfunction in endotoxemia [9, 49, 50]. Importantly, S100A8/A9 binding to TLR-4 has been shown to have a cardio-depressant effect in myocardial infarction by direct inhibition of mitochondrial function in cardiomyocytes [51]. In line with these findings, we show here that the ABR-238901 treatment rescues cardiac mitochondrial dysfunction induced by endotoxemia. In contrast to mRNA silencing, S100A8/A9 blockade with a small-molecule inhibitor is more clinically relevant due to the immediate effect, broad systemic outreach, treatment flexibility, costs and accessibility. Importantly, we show that delayed ABR-238901 administration has an immediate beneficial effect on cardiac dysfunction even in the setting of already established disease, and is more effective than Dexamethasone. Glucocorticoid therapy is one of the few viable anti-inflammatory therapies against exacerbated immune activation in various disease settings, and has also been used for treatment of SARS-CoV-2 patients. However, the use of glucocorticoid therapy during sepsis is controversial, and a recent panel provided only a weak recommendation for their use in sepsis patients [52]. The main caveats of using glucocorticoids in sepsis are the potent and long-lasting immunosuppressive effects which impair the ability of the host to fight the bacterial invasion. In contrast, our small-molecule anti-S100A8/A9 treatment is fast-acting, efficiently reverses cardiac dysfunction, and can be interrupted as soon as the patients show signs of cardiac recovery. In our previous studies in mice with myocardial infarction, we found that the anti-inflammatory effects of short-term ABR-238901 treatment lead to improved cardiac function [32]. In contrast, long-term S100A8/A9 inhibition impairs myocardial repair and counteracts the initial beneficial effects of the treatment [53]. In the context of sepsis, in order to prevent immunosuppression, we advocate maintaining the use of S100A8/A9 blockade to the shortest possible period, until anti-inflammatory effects and cardiovascular stabilization are observed.

### Study limitations

Our study has a few limitations which are important to consider. We provide evidence for a connection between high plasma S100A8/A9 and LV dysfunction at ICU admission in patients with severe sepsis. These results have to be interpreted with due caution, as echocardiographic evaluation of LV function in sepsis is difficult and is highly influenced by the reduced afterload due to vasoplegia [54]. To address this caveat, we used a combined evaluation of multiple parameters of LV function, as recommended by the PRICES consensus [30]. However, this approach has yet to be validated in larger trials. Secondly, the cohort is under-dimensioned to be able to assess potential associations between S100A8/A9, cardiac dysfunction and mortality. As discussed above, the relationship between S100A8/A9 and mortality in sepsis has previously been demonstrated by other studies including larger populations [20–23]. It is unlikely that cardiac dysfunction is the sole link between elevated S100A8/A9 and mortality, but it might be an important contributor. The potent systemic anti-inflammatory effects of the treatment, alongside the previously-demonstrated ability to prevent NETs release and lung inflammation in the CLP mouse model of abdominal sepsis witness a broader therapeutic effect that might contribute to reduced mortality and improved patient prognosis [46, 47]. Thirdly, we chose to test the treatment in the LPS-driven disease model, as LPS is a component of Gram-negative bacterial wall and the most potent stimulator of innate immune responses in infections. It remains to be determined if similar effects can be seen in Gram-positive bacteria, viral or fungi infections. The previously demonstrated anti-inflammatory and disease protective effects of ABR-238901 in the CLP mouse model of multibacterial sepsis demonstrate that these effects are not model-dependent [46, 47]. Lastly, there are important differences in the patterns of cardiac dysfunction in experimental sepsis models and sepsis patients. While LVEF and stroke volumes are severely depressed in our mouse model, sepsis patients have highly heterogeneous patterns of LVEF and LV volumes [6]. In sepsis patients with depressed LVEF, cardiac dilation may also be present as a compensatory mechanism to preserve stroke volume [54–56]. Due to these important differences, the potential therapeutic effects of S100A8/A9 blockade on cardiac dysfunction in sepsis demonstrated herein cannot be directly extrapolated to the clinical scenario. However, the potent systemic anti-inflammatory effects of the treatment and the previously demonstrated benefits in other sepsis models provide encouraging evidence for treatment efficacy in view of future clinical development [26, 47].

## Conclusions

We demonstrate that high S100A8/A9 levels are associated with acute LV dysfunction in patients with severe sepsis, and that pharmacologic S100A8/A9 blockade with the small-molecule inhibitor ABR-238901 can mitigate myocardial dysfunction in endotoxemia. The treatment potently reduced the systemic levels of inflammatory cytokines and chemokines, dampened inflammation and improved mitochondrial function in the myocardium. ABR-238901 was able to rapidly reverse already established systolic LV dysfunction, which is likely to be present at hospital admission in severe sepsis patients with high S100A8/A9 levels. In contrast, Dexamethasone did not improve cardiac function in this context. Our results identify S100A8/A9 as a potential marker of cardiac dysfunction in sepsis patients and promote systemic S100A8/A9 blockade as a viable treatment to ameliorate SIMD and improve patient prognosis. S100A8/A9 (calprotectin) is already used in the clinic to detect exacerbations of inflammatory bowel disease, and ELISA- or turbidimetric-based methods for measuring calprotectin in plasma or serum are already established in clinical laboratories. Importantly, earlier generations of S100A8/A9 blockers including laquinimod (ABR-215062), tasquinimod (ABR-215050) and paquinimod (ABR-215757), have been or are currently undergoing clinical testing for treatment of inflammatory diseases and cancer [57]. Laquinimod has shown clinical efficacy in Crohn’s disease and multiple sclerosis, paquinimod has been tested in systemic sclerosis, and tasquinimod is undergoing a clinical trial in multiple myeloma (ClinicalTrials.gov: NCT04405167) [58–60]. Here, we provide proof of concept that S100A8/A9 blockade reduces inflammation and cardiac dysfunction in endotoxemia. Our findings open the way for future clinical testing or re-purposing of these S100A8/A9 blockers for the treatment of patients with severe sepsis.

### Supplementary Information

**Additional file 1: Figure 1** Cardiac immune cell infiltration during endotoxemia. Numbers of infiltrating cardiac immune cell populations in naïve mice and at 24 h post-LPS in endotoxemic mice treated with PBS or ABR-238901 at 0 h and 6 h. Statistical differences between the groups were tested with 1-way ANOVA with Fisher’s LSD Test or Kruskal–Wallis test for nonparametric data, normality was assessed with Shapiro–Wilk test * indicates comparison between naïve mice and mice treated with LPS + PBS, **P < 0.01, ***P < 0.001; LPS, Lipopolysaccharide; PBS, Phosphate Buffered Saline; ABR, ABR-238901; NK, Natural killer cell; DCs, Dendritic cells. Data is represented as mean ± SD. N = 4–5 per group.

**Additional file 2: Figure 2** Cardiac mitochondrial function during endotoxemia. **A** Experimental layout. **B**–**E** Interfibrillar mitochondria (IFM) function. **B** Citrate synthase activity. **C** Mitochondrial yield. **D** Oxygen consumption rate (OCR) after incubation with respiration substrates feeding complex I (malate + glutamate), either in the absence (state 2) or presence (state 3) of ADP. **E** Oxygen consumption rate after incubation with respiration substrates feeding complex II (succinate + rotenone). **F**, **G** Reactive oxygen species (ROS) production levels in subsarcolemal (SSM) and IFM mitochondria. Differences between the three groups were tested using 1-way ANOVA with Fisher’s LSD test. Normality assessment was performed with Shapiro–Wilk test. LPS, lipopolysaccharide; ABR, ABR-238901; OCR, Oxygen consumption rate. Data are represented as mean ± SD from N = 4–7 per group.

## Data Availability

All data underlying this article are available in the article and in its online supplementary material.

## Abbreviations

IFM: Interfibrillar mitochondria
IFN-γ: Interferon gamma
IL-1β: Interleukin-1 beta
LVEF: Left ventricular ejection fraction
LPS: Lipopolysaccharide
RAGE: Receptor for Advanced Glycation End-products
SAPS: Simplified Acute Physiology Score
SOFA: Sequential Organ Failure Assessment
SSM: Subsarcolemmal mitochondria
TLR-4: Toll-like receptor 4
TNF-α: Tumor necrosis factor alpha
