# Supplementary figures and images for: Therapeutic S100A8/A9 blockade inhibits myocardial and systemic inflammation and mitigates sepsis-induced myocardial dysfunction
Source: Crit Care. 2023 Sep 29;27:374. doi: 10.1186/s13054-023-04652-x (PMC10540409; doi:10.1186/s13054-023-04652-x)

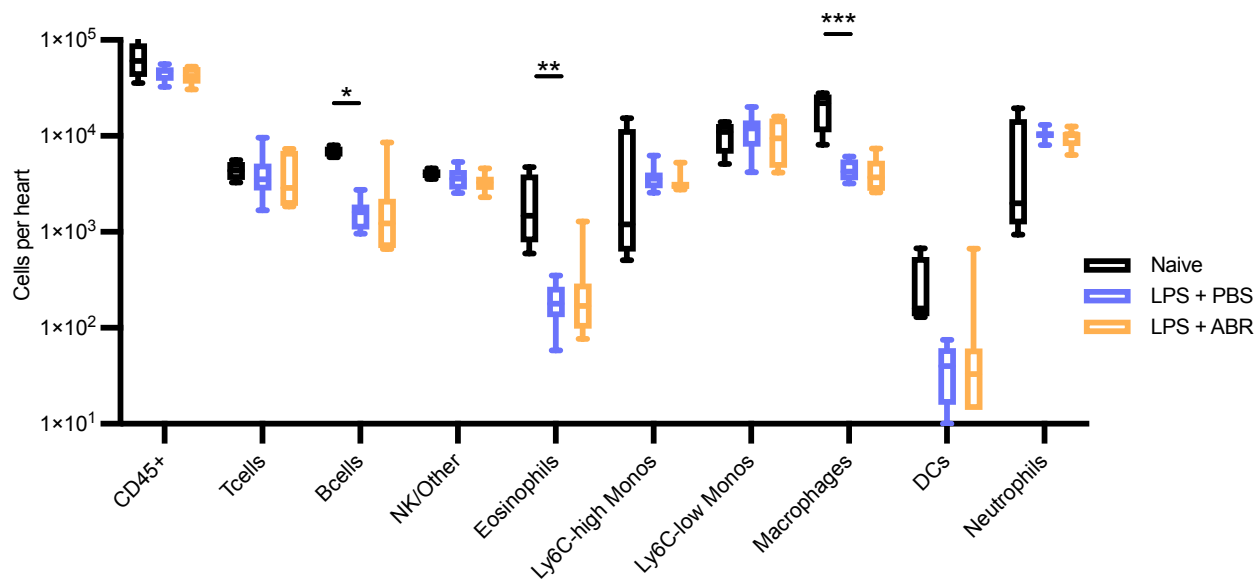

Supplement: Supplementary file 1 — Additional file 1: Figure 1 Cardiac immune cell infiltration during endotoxemia. Numbers of infiltrating cardiac immune cell populations in naïve mice and at 24 h post-LPS in endotoxemic mice treated with PBS or ABR-238901 at 0 h and 6 h. Statistical differences between the groups were tested with 1-way ANOVA with Fisher’s LSD Test or Kruskal–Wallis test for nonparametric data, normality was assessed with Shapiro–Wilk test * indicates comparison between naïve mice and mice treated with LPS + PBS, **P < 0.01, ***P < 0.001; LPS, Lipopolysaccharide; PBS, Phosphate Buffered Saline; ABR, ABR-238901; NK, Natural killer cell; DCs, Dendritic cells. Data is represented as mean ± SD. N = 4–5 per group. [file 13054_2023_4652_MOESM1_ESM.pdf]

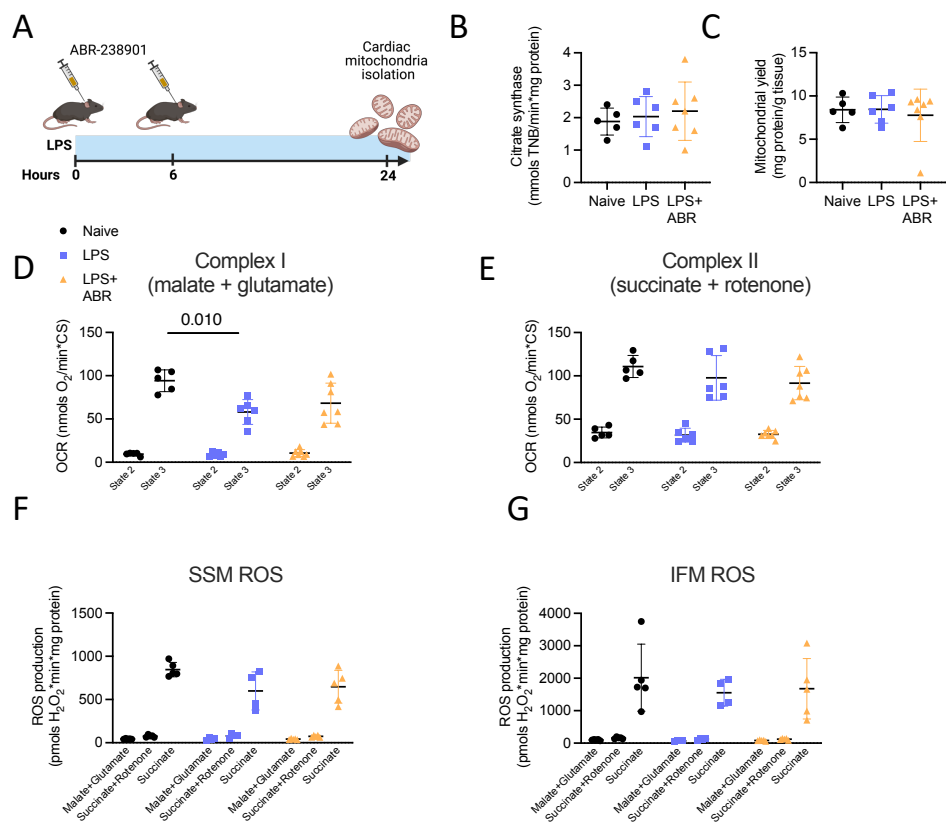

Supplement: Supplementary file 2 — Additional file 2: Figure 2 Cardiac mitochondrial function during endotoxemia. A Experimental layout. B–E Interfibrillar mitochondria (IFM) function. B Citrate synthase activity. C Mitochondrial yield. D Oxygen consumption rate (OCR) after incubation with respiration substrates feeding complex I (malate + glutamate), either in the absence (state 2) or presence (state 3) of ADP. E Oxygen consumption rate after incubation with respiration substrates feeding complex II (succinate + rotenone). F, G Reactive oxygen species (ROS) production levels in subsarcolemal (SSM) and IFM mitochondria. Differences between the three groups were tested using 1-way ANOVA with Fisher’s LSD test. Normality assessment was performed with Shapiro–Wilk test. LPS, lipopolysaccharide; ABR, ABR-238901; OCR, Oxygen consumption rate. Data are represented as mean ± SD from N = 4–7 per group. [file 13054_2023_4652_MOESM2_ESM.pdf]
